# Automated elucidation of crystal and electronic structures in boron nitride from X-ray absorption spectra using uniform manifold approximation and projection

**DOI:** 10.1038/s41598-025-18580-z

**Published:** 2025-11-10

**Authors:** Reika Hasegawa, Arpita Varadwaj, Alexandre Lira Foggiatto, Masahito Niibe, Takahiro Yamazaki, Masafumi Horio, Yasunobu Ando, Takahiro Kondo, Iwao Matsuda, Masato Kotsugi

**Affiliations:** 1 Department of Material Science and Technology, Tokyo University of Science, 6-3-1, Niijuku, Katsushika, Tokyo 125-8585 Japan; 2 Institute for Solid State Physics, The University of Tokyo, Kashiwanoha 5-1-5, Kashiwa, Chiba 277-8581 Japan; 3 Institute of Integrated Research, Institute of Science Tokyo, Yokohama, Kanagawa 226-8501 Japan; 4 Institute of Pure and Applied Sciences, University of Tsukuba, Tsukuba, Ibaraki 305-8573 Japan

**Keywords:** Electronic structure, Computational chemistry, Structure prediction, Electronic materials, Photocatalysis

## Abstract

X-ray absorption spectroscopy (XAS) provides valuable information on the structure and electronic states of materials. Its spectral profiles are complex, influenced by crystal structure and defects, and require extensive expertise for conventional analysis. This study presents an automated XAS analysis approach using various dimension reduction methods to characterize crystal and electronic structure. We show that, Uniform Manifold Approximation and Projection (UMAP), outperforms traditional methods such as Principal Component Analysis (PCA) and Multidimensional Scaling (MDS) in capturing the key features of complex spectra. Applying UMAP to simulated XAS spectra of boron nitride (BN), high-dimensional data can be precisely classified by crystal system, defect type, and structural dimensionality. By correlating UMAP-derived embeddings with electronic states, we identify nontrivial electronic properties. The successful application of this model to experimental data demonstrates its potential for autonomous structural identification, offering new possibilities for data-driven materials design and advancing novel material development.

**Supplementary Information:**

The online version contains supplementary material available at 10.1038/s41598-025-18580-z.

## Introduction

X-ray absorption spectroscopy (XAS) is a vital tool for elucidating material properties by providing detailed information on crystal structures, electronic states, and functional characteristics^1–3^. In particular, the XAS spectra at the B K-edge in boron compounds exemplify the complexity of spectral features, which vary with crystal system^4^, layer number, and defect configuration. These spectral variations are intimately related to functional properties such as luminescence and electronic performance. Boron compounds are especially attractive owing to their high carrier density^5,6^, chemical stability^7^, low weight, and minimal environmental impact. Consequently, they have garnered significant attention for applications in advanced semiconductor devices^8–10^, IoT communication devices^11^, energy storage^12,13^, and catalysis^14–16^. In these applications, a deep understanding of the electronic state is crucial for rational material design^17^. Recently, various data-driven analysis methods that integrate data science with measurement science have emerged as powerful tools for efficiently analyzing and interpreting measurement data, including spectral features that reflect electronic structure. By leveraging these approaches, researchers can gain deeper insights into the complex electronic and structural properties of boron compounds, facilitating their optimization for targeted applications^18^.

Historically, the properties of boron compounds have been analyzed through both computational and experimental approaches^10,19^. First-principles calculations have been employed to determine stable crystal structures to calculate band dispersions and densities of states (DOS), and to simulate XAS spectra via methods such as the Supercell Core Hole (SCH) approach. It has been observed that modifications such as transitioning among layered BN, cubic BN (c-BN), and wurtzite BN (w-BN), or introducing defects and dopants lead to significant changes in bonding characteristics and, consequently, to complex variations in the XAS spectra^20^. Detailed analyses of peak positions, intensity ratios, and fine spectral structures have provided insight into the balance between in-plane $\upsigma$-bonds and out-of-plane $\uppi$-bonds, as well as into the chemical bonding at B and N sites. In particular, vacancies and impurities in boron nitride have been extensively studied^21–24^, because it significantly affects its device performance^25–28^ and its catalytic activity^29,30^. However, such analyses traditionally require extensive expert knowledge and considerable manual effort, particularly when large datasets are examined through visual inspection^31^. Establishing a systematic and objective linkage between XAS spectra and underlying material properties remains challenging.

Experimentally, synchrotron-based XAS measurements have long been used to investigate the local structural and electronic environments of materials. Even subtle structural variations and defect-induced changes are reflected in the XAS spectra^32^. However, sample preprocessing and background subtraction often introduce additional complexity, necessitating advanced analytical skills and resulting in subjective interpretations. This subjectivity, coupled with the labor-intensive nature of manual spectral comparisons, hampers the clear and rational correlation of spectral features with specific structural or electronic characteristics, particularly in the context of experimentally synthesized materials where the presence and nature of defects or local structural variations are less characterized beforehand and where prior reference spectra may be unavailable or incomplete.

To address these challenges, we propose an automated approach that employs machine learning to analyze XAS spectra and extract information on both crystal structure and electronic state. Our method is founded on unsupervised manifold learning, which is well suited to handle the intrinsic complexity of XAS data. Although the excitation process in XAS involves nonlinear effects, the underlying physics can be described by a relatively small number of matrix operations. This observation suggests that the effective dimensionality of XAS spectral data is low, supporting the application of the manifold hypothesis. Accordingly, we explore both linear and nonlinear dimensionality reduction techniques such as principal component analysis (PCA)^33^, multidimensional scaling (MDS)^34^, the t-distributed Stochastic Neighbor Embedding (t-SNE)^35^, and Uniform Manifold Approximation and Projection (UMAP)^36^ to project high-dimensional spectral data onto a lower-dimensional space. These unsupervised and interpretable approaches^37^ enable automated, objective, and scalable analysis, significantly reducing subjectivity and facilitating the integration of computational and experimental data.

While the computational generation of XAS spectra involves manual setup and convergence tuning, the subsequent analysis process including spectral clustering, dimensionality reduction, and trend extraction is carried out using unsupervised machine learning algorithms. This approach minimizes subjective interpretation, supports reproducibility, and can be extended to other systems without requiring retraining or feature engineering.

The application of unsupervised learning methods, such as PCA, t-SNE, and various clustering algorithms to spectroscopic data has gained increasing attention in recent years. These methods have been used to uncover latent patterns in IR, Raman, and X-ray spectroscopies, facilitating classification of materials based on their local environments or functional groups. For instance, PCA has been applied to denoise and extract key spectral components, while t-SNE and UMAP have aided in visualizing similarities across large spectral datasets^38–41^. However, most existing studies have focused on organic molecules^42^ or well-known material systems^43^, and they rarely benchmark the performance of multiple unsupervised algorithms across both simulated and experimental datasets. Furthermore, the physical interpretability of such dimensionality reduction techniques particularly in relation to crystal structures, electronic states, and defect configurations remained underexplored.

In the context of XAS for light elements such as boron and carbon, automated spectral analysis remains especially limited. These K-edge spectra are inherently complex, with broad and overlapping peaks that are highly sensitive to subtle structural and electronic changes. A recent first-principles study has successfully attributed B K-edge spectral features in hexagonal boron nitride (h-BN) to specific defect types. For instance, intermediate-energy peaks have been linked to oxygen-related impurity configurations through charge transfer and local bonding analysis^44^. This approach, however, relies heavily on detailed electronic structure calculations and manual spectral interpretation. To the best of our knowledge, no prior study has established a unified framework for machine learning-based classification of such spectral features, nor systematically compared dimensionality reduction techniques in this context.

Towards this end, in this work, we perform a systematic comparison of dimensionality reduction techniques including the linear method PCA, the nonlinear embedding method t-SNE, and the manifold learning UMAP on a dataset of B K-edge XAS spectra primarily generated by first-principles simulations of BN systems with controlled structural variations, including h-BN, c-BN, w-BN, and structures with point defects. Three experimental spectra were also included and successfully mapped to their corresponding clusters. Our results show that UMAP, in particular, provides a robust and interpretable low-dimensional representation that clusters spectra according to structural motifs and defect configurations. By linking these embeddings with physical descriptors such as local bonding environments and density of states, we demonstrate a pathway for rapid, objective, and scalable interpretation of complex XAS datasets especially for lightweight inorganic materials where conventional analysis remains challenging.

**Fig. 1:**
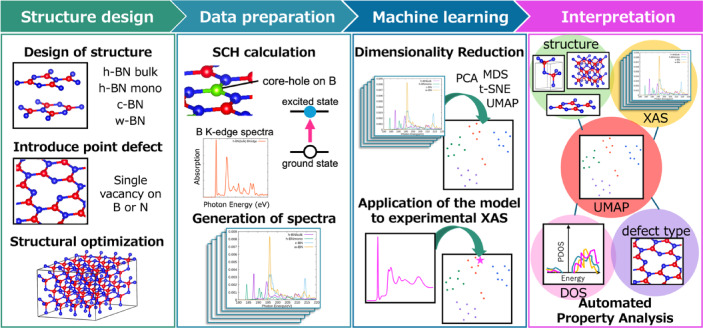
Workflow of the present study. Initially, structural optimization was performed for various BN systems, and high-dimensional XAS spectra along with DOS spectra were generated using SCH calculations. Subsequently, unsupervised machine learning techniques were employed to reduce the XAS spectra to a low-dimensional space, thereby extracting the intrinsic information contained within the high-dimensional data. The clustering results were then analyzed to elucidate the relationships among the XAS spectra, crystal structures, electronic states, and atomic defects. Finally, experimental data were projected into the low-dimensional space to explore the potential for integrated computational and experimental material identification.

Figure 1 illustrates the overall workflow of our study. First, representative BN crystal structures (h-BN, c-BN, and w-BN) and systems with point defects are calculated using first-principles methods. After structural optimization, XAS spectra and electronic states are computed using the SCH method under tight convergence criteria, ensuring high model reliability across a dataset that spans different crystal systems, defect types, and layer dependencies. The resulting spectral data are then subjected to manifold learning analysis, with the goal of classifying complex spectral features and evaluating clustering accuracy with respect to known variations in structure and defects. Furthermore, by correlating the reduced-dimensional representations with electronic properties such as DOS and bonding configurations, we aim to provide a deeper physical interpretation of the machine learning results. Finally, we validate the effectiveness of our approach by applying the model derived from computational data to experimental XAS spectra, thereby providing a guideline for characterizing various material properties.

This study aims to bridge the gap between XAS spectral data and the underlying structural and electronic properties through an unsupervised machine learning framework emphasizing high interpretability of pattern discovery in material properties such as lattice structure and electronic states. This approach promises to streamline the analysis process, reduces manual overhead in data interpretation and to provide a robust pathway for the rational design of advanced functional materials.

## Results and discussion

**Fig. 2:**
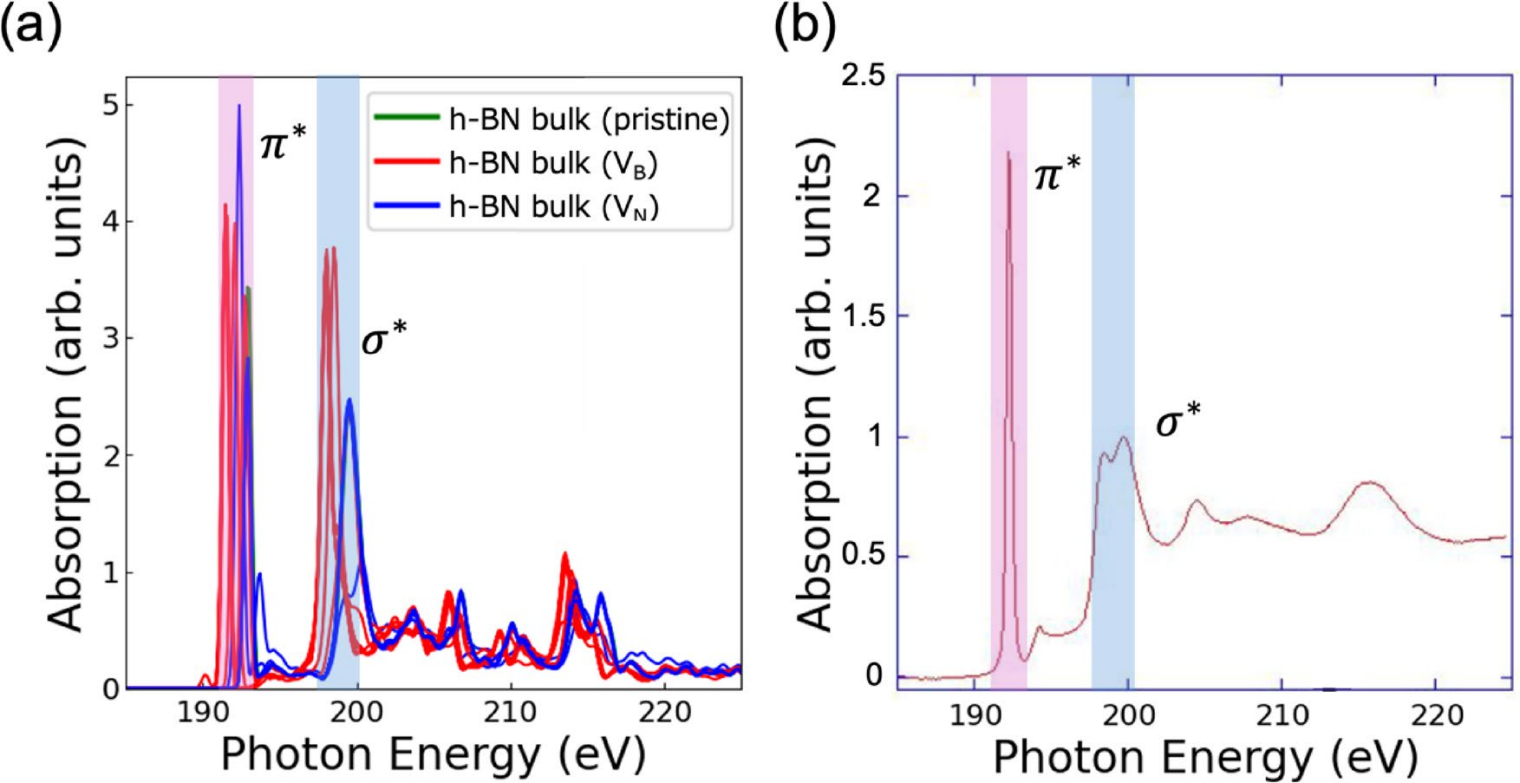
(**a**) Computed B K-edge XAS spectra of h-BN bulk and its point-defect configurations, including pristine h-BN (green), boron vacancy (V_B_, red), and nitrogen vacancy (V_N_, blue). The spectra were obtained from first-principles calculations using a core-hole approach. Subtle variations in peak positions and intensities, particularly in the π* and σ* regions, reflect the local electronic perturbations introduced by point defects, and (**b**) Experimental h‑BN XAS spectra. The ${\pi }^{*}$ peak appears around 192 eV, the ${\sigma }^{*}$ peak around 199 eV, and fine structure is observed near 215 eV, with peak positions and widths in reasonable agreement between calculation and experiment. This validation confirms the accuracy of the first-principles calculated XAS spectra.

To validate the 68 X-ray absorption spectra (XAS) obtained from first-principles calculations, we conducted a detailed comparative analysis, focusing primarily on the 17 h-BN bulk spectra (B K‐edge) included in the dataset. These calculated spectra were directly compared with experimentally measured h-BN XAS spectra (B K‐edge) (see Fig. 2). The h-BN sample was purchased from JFE Mineral & Alloy Company, Ltd. XAS measurements were performed at beamline BL-09A at the NewSUBARU^45^. The spectra were acquired by total electron yield (TEY) method^46^. The comparison revealed a reasonably good correspondence between the calculated and experimental spectra. In particular, both peak positions and peak widths demonstrated excellent agreement, with the ${\uppi }^{*}$ peak near 192 eV, the ${\upsigma }^{*}$ peak near 199 eV, and the spectral features observed around 215 eV closely aligning between the calculated and experimental results. These features, which reflect the intrinsic electronic structure of h-BN, strongly support the accuracy of our computational approach.

Further validation was carried out for the other structural configurations. For h-BN mono^20,47^, c-BN^48^, and w-BN^48^, the calculated spectra showed matching peak positions and widths when compared with their respective experimental spectra. This comprehensive analysis demonstrates that the XAS spectra derived from our first-principles calculations faithfully replicate experimental observations with high precision.

These findings provide robust support for the validity of our computational methodology indicating that the model accurately captures the electronic state of BN. In conclusion, the theoretically calculated XAS spectra utilized in this study have been confirmed as reliable input data, thereby reinforcing the subsequent analyses and discussions.

**Fig. 3:**
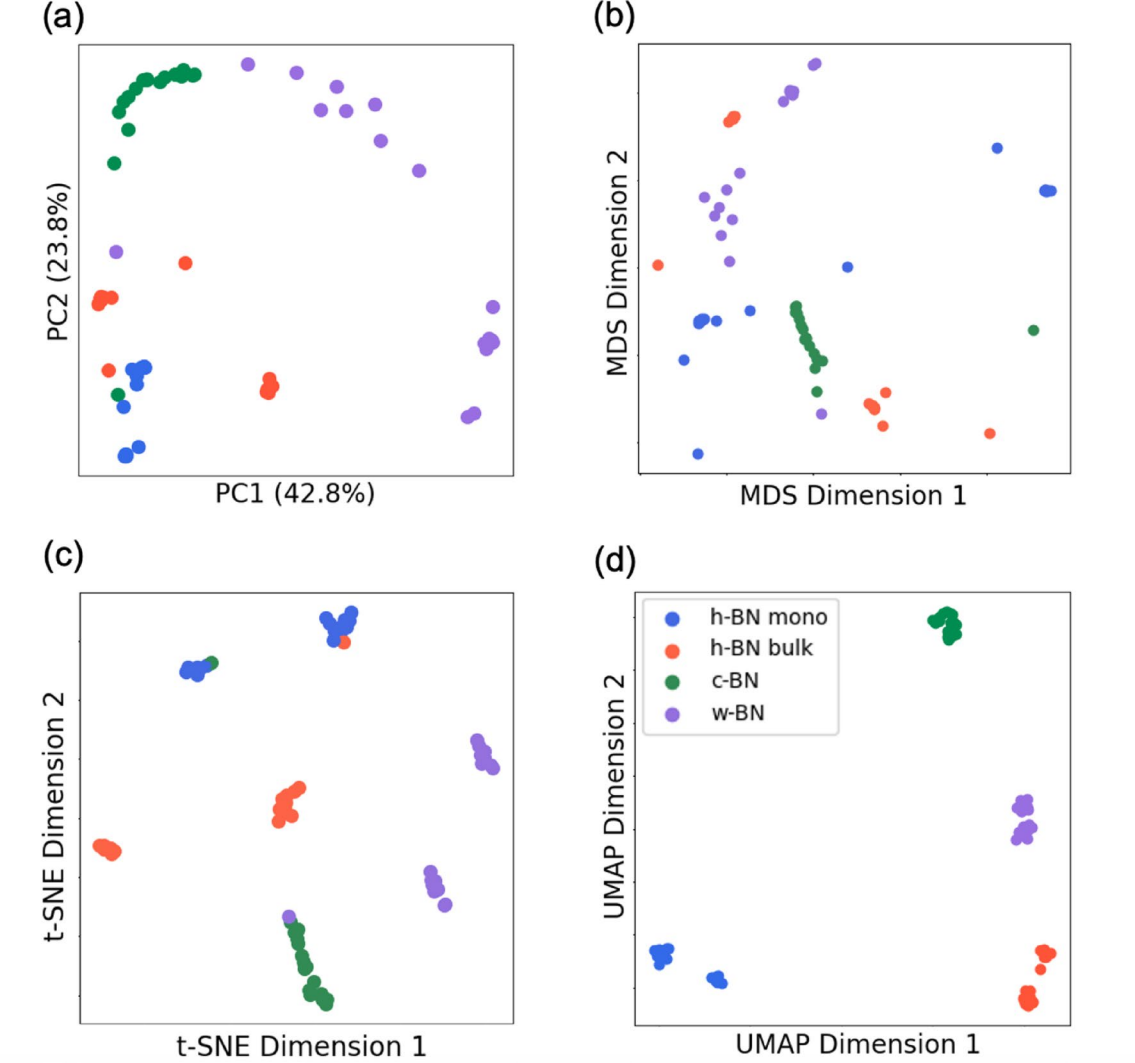
Two-dimensional embeddings of the XAS spectra obtained using (**a**) PCA, (**b**) MDS, (**c**) t-SNE, and (**d**) UMAP. In these plots, h-BN monolayer is colored blue, h-BN bulk orange, c-BN green, and w-BN purple. (**a**) PCA failed to clearly cluster h-BN bulk and h-BN monolayer, and the cumulative contribution of PC1 and PC2 was relatively low at 66.6%. (**b**) Similarly, MDS employing cosine similarity did not achieve clustering by crystal system, as evidenced by a high stress value of 0.150. (**c**) t-SNE, while generally clustering the data according to crystal system, exhibited some misclassification among certain systems. (**d**) In contrast, UMAP successfully and distinctly clustered the spectra by crystal system, particularly discriminating between the spectrally similar h-BN bulk and h-BN monolayer.

We next subjected the 68 XAS spectra to dimensionality reduction using PCA, MDS, t-SNE, and UMAP. Rather than relying on manual comparison of raw spectra, our method uses low-dimensional embeddings generated by these unsupervised algorithms. These projections map the high-dimensional spectral data into a 2D hyperplane, where clustering patterns emerge based on the spectral similarity. As a result, groupings are data-driven and reproducible, independent of visual bias. Among the methods tested, PCA failed to adequately cluster the XAS spectra by structure (Fig. 3a), with the cumulative contribution of PC1 and PC2 amounting to 66.6%. Notably, PCA was unable to differentiate between the h-BN bulk and monolayer spectra, whose overall shapes are very similar. Examination of the eigenvectors for the first and second principal components (Fig. S1) reveals that both are dominated by the energy region associated with $\upsigma$ bonding, whereas the energy range corresponding to the $\uppi$ bonding peak contributes little. This observation aligns with the fact that c-BN and w-BN characterized primarily by their ${\upsigma }^{*}$ peaks are reasonably well clustered, while the h-BN bulk and monolayer, which also exhibit distinct ${\uppi }^{*}$ features, are not separated. Thus, PCA appears to capture only a subset of the complex spectral features of BN, failing to account for the fine structure associated with $\uppi$ bonding. These results suggest that linear methods such as PCA are ill-suited to fully capture the nonlinear variations present in the complex spectral data of BN.

MDS, with cosine similarity as the distance metric (Fig. 3b), exhibited similar limitations. The structural clusters were poorly separated, with two-dimensional materials and bulk samples failing to segregate clearly. Furthermore, the obtained stress value of 0.150 is quite high, indicating substantial information loss during the mapping process. This high stress value correlates with the inability to cluster h-BN monolayer and bulk both of which share similar peak features as well as the overall failure to distinguish between two-dimensional and bulk structures. Although MDS utilizes a nonlinear cosine similarity metric, it primarily emphasizes points with high variance, leading to significant loss of detailed spectral information during the low-dimensional projection.

t-SNE approach revealed clusters corresponding to different crystal systems; however, some plots from distinct systems overlapped (Fig. 3c). In addition, each crystal system cluster split into two major groups. Upon closer inspection, the spectra that fell into these mixed clusters were all computed with a core hole introduced on atoms adjacent to defects, and these spectra exhibited marked deviations in shape compared to the others (Fig. S2). This suggests that the environmental perturbations induced by nearby defects result in irregular spectral changes that t-SNE could not adequately account for. Although t-SNE is effective at elucidating local structure in high-dimensional data, it sometimes fails to capture the global structure accurately^49^. In our dataset, where peak positions, widths, and defect-induced variations differ significantly across crystal systems, successful clustering requires accurate representation of both local and global structures. t-SNE’s inability to concurrently preserve these aspects likely led to some spectra being misclassified among crystal systems.

In contrast, UMAP yielded distinctly concentrated clusters corresponding to each crystal system (Fig. 3d). 

Notably, UMAP accurately separated two-dimensional materials (h-BN) from bulk systems (c-BN and w-BN) and further distinguished between h-BN bulk and monolayer, suggesting that nontrivial structural dimensionality differences can be identified directly from the XAS spectra. The excellent performance of UMAP can be attributed to its foundation on the manifold hypothesis, which enables it to map the original high-dimensional spectral data into a low-dimensional space with minimal loss of key features. Given that the XAS spectra are computed from a relatively small number of matrix elements and involve nonlinear excitation processes, they conform well to the manifold hypothesis. Therefore, UMAP is particularly well suited for clustering the complex, nonlinear XAS spectral data while preserving both local and global structures.

Our dimensionality reduction results indicate that UMAP could be one of the appropriate methods for analyzing XAS spectra. The intrinsic features of XAS spectra where the density of states is represented as a probability density and the absorption process itself is probabilistic render them amenable to statistical machine learning. Additionally, the underlying excitation process involves non-diagonal matrix elements, supporting the relevance of the manifold hypothesis. Given these characteristics, nonlinear dimensionality reduction techniques could be effective. UMAP, as a representative method of manifold learning and nonlinear dimensionality reduction, offers several mathematical advantages that make it particularly effective for analyzing XAS spectra.

First, UMAP is inherently designed for data that conform to the principles of manifold learning, ensuring that low-dimensional embeddings preserve the essential structure of high-dimensional data. Second, by leveraging Riemannian geometry, UMAP maintains inter-data distances and preserves fine structural details within spectral data, which is crucial for accurately capturing variations in X-ray absorption spectroscopy (XAS) spectra. Third, its use of fuzzy topological representations ensures that local relationships among data points remain intact, preventing the loss of important structural information during dimensionality reduction. Finally, UMAP integrates cross-entropy optimization with stochastic gradient descent (SGD), enabling it to retain the global structure of the data while achieving an effective lower-dimensional representation.

These properties allow UMAP to effectively reduce dimensionality while preserving both local and global structures. Given the low effective dimensionality of the XAS spectra and their nonlinear nature, UMAP is exceptionally well matched to these data. Furthermore, because both DOS and XAS can be treated probabilistically and cross-entropy serves as a robust metric for quantifying spectral differences UMAP holds significant potential for broader applications in spectroscopic analysis involving probabilistic excitation processes.

**Fig. 4:**
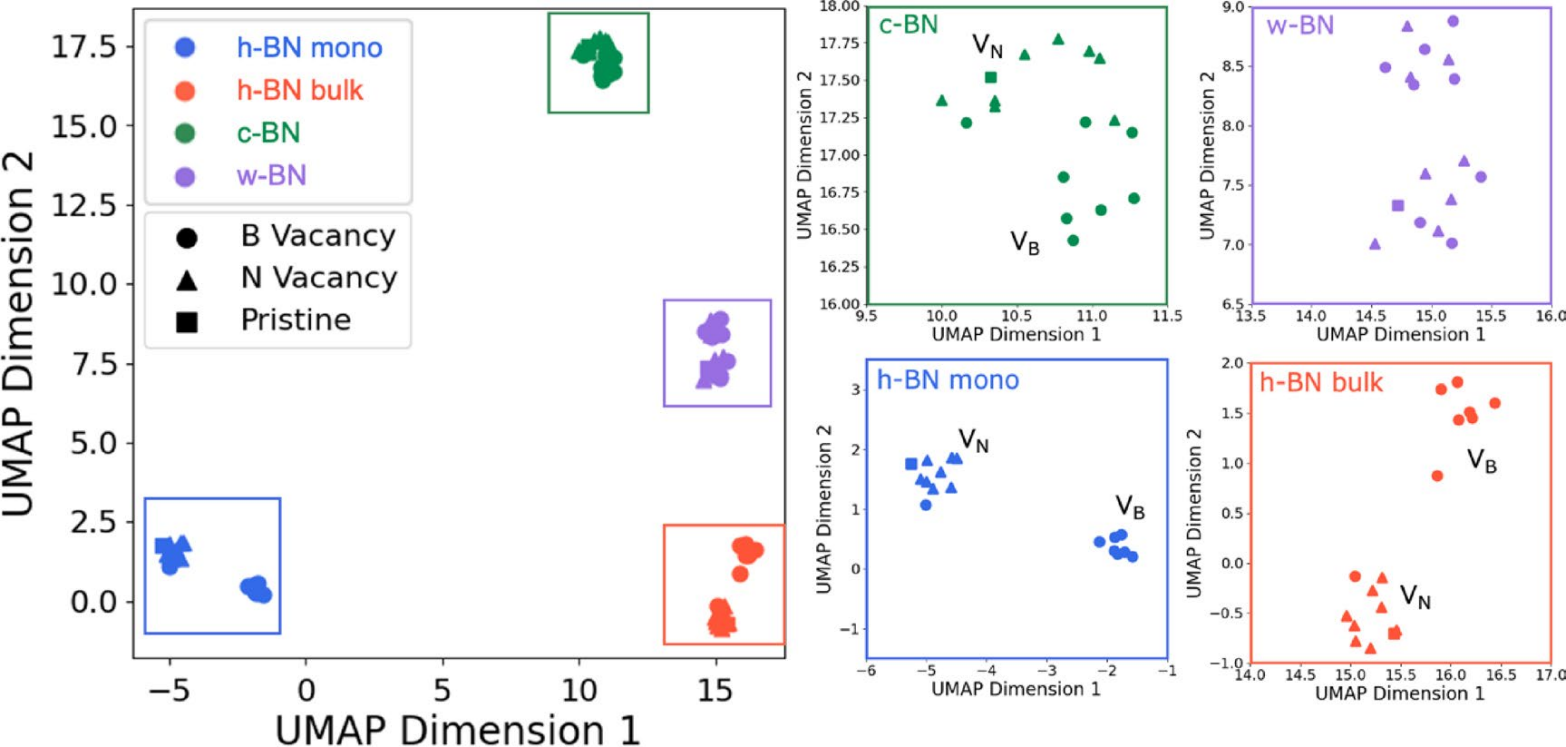
Enlarged views of the UMAP embeddings of the XAS spectra, separated by crystal system. In these expanded plots, data points are further distinguished by defect type: VB is represented by circles, VN by triangles, and pristine by squares. The enlarged views reveal that h‑BN bulk, h‑BN monolayer, and c‑BN each exhibit clustering based on defect type, whereas the two clusters observed for w‑BN are not distinguished by defect type.

Additionally, for the pristine, V_B_, and V_N_ spectra were distinguished by marker shape on the UMAP plot (Fig. 4),

the h‑BN monolayer and bulk spectra showed that V_B_ and V_N_ formed relatively distinct clusters. This successful clustering by both crystal system and defect type underscores the sensitivity of UMAP to subtle spectral variations. In contrast, for c‑BN, the boundary between V_B_ and V_N_ was less distinct; however, inspection of the original spectra revealed only minor differences in spectral shape (Fig. S3). While a few data points might be considered misclassified, their original spectral profiles were very similar, suggesting that UMAP effectively captures even slight changes in the XAS spectral features. Thereby, UMAP emerges as the most suitable method for the analysis and visualization of complex XAS spectra. Its ability to retain both the local and global structures of the data makes it an invaluable tool for clustering by crystal system and defect type, thereby revealing subtle but critical differences in the spectra that may be obscured by other dimensionality reduction techniques.

**Fig. 5:**
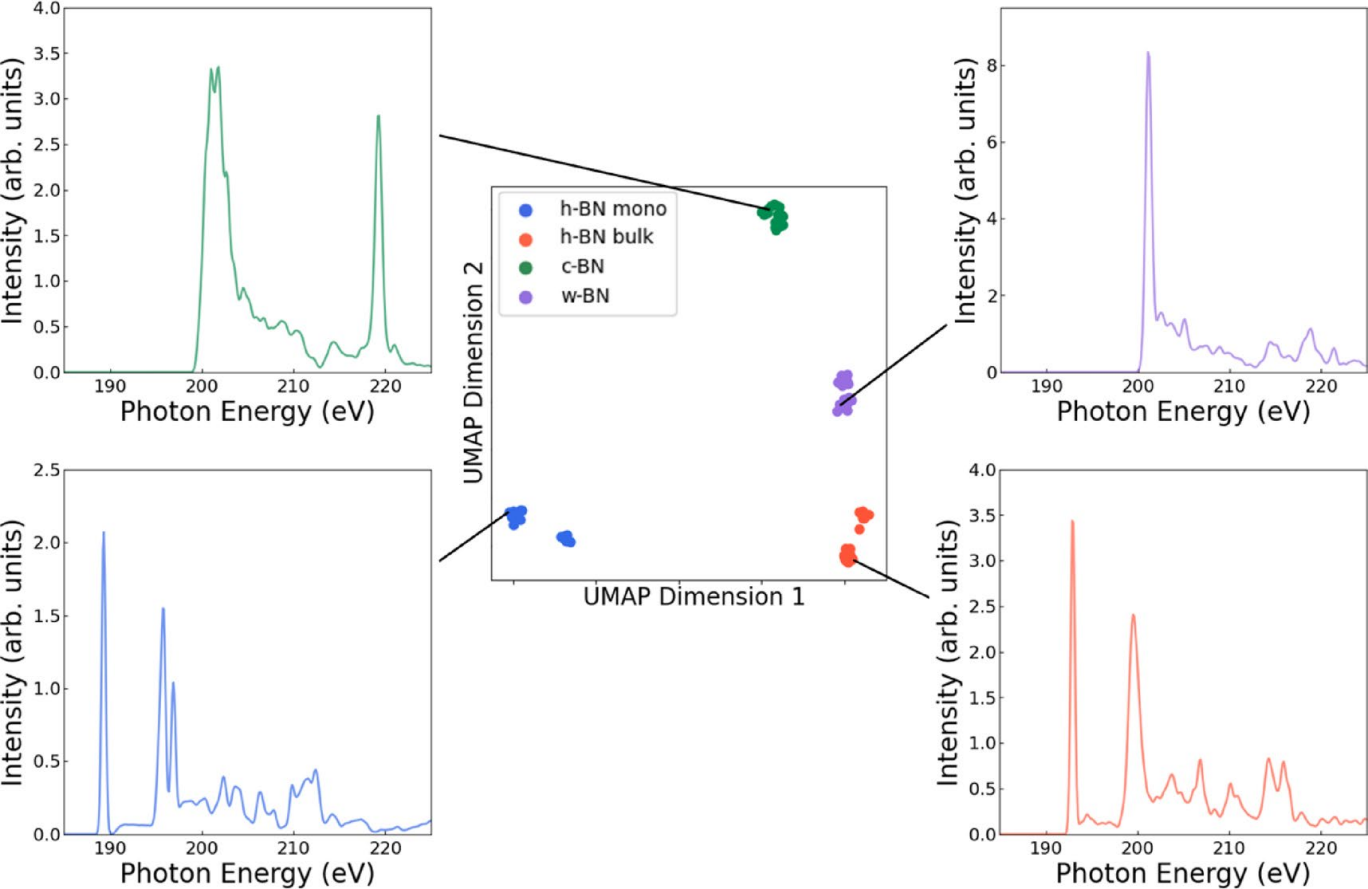
Representative XAS spectra corresponding to each UMAP-identified cluster. Each spectrum was selected from a data point closest to the cluster centroid in the UMAP space. Distinct spectral features such as peak positions, intensity variations, and edge shapes reflect the differences that drive the clustering. These variations help interpret how the unsupervised algorithm separates different structural or chemical forms of boron nitride.

To better understand the spectral features driving the UMAP clustering, we extracted representative XAS spectra from each cluster, as shown in Fig. 5.

For each group, a data point closest to the cluster centroid in the UMAP space was selected, and its corresponding XAS spectrum was plotted. The figure reveals key differences such as subtle shifts in π* and σ* peak positions, variations in peak intensity, and overall spectral broadening that underpin the observed clustering patterns. These variations arise from fundamental physical differences in the boron nitride systems. Peak shifts indicate changes in the local bonding environment induced by different defect types, differences in intensity point to variations in orbital hybridization, and spectral broadening is linked to local disorder. These physical distinctions drive the formation of separate clusters in the UMAP space, showing that the method captures meaningful structural and electronic variations rather than just statistical similarity. Thus, the visualization confirms that UMAP not only clusters based on global spectral correspondence but also detects fine differences associated with different phases and defect types in boron nitride, reinforcing its utility as a powerful tool for interpreting complex XAS datasets.

**Fig. 6:**
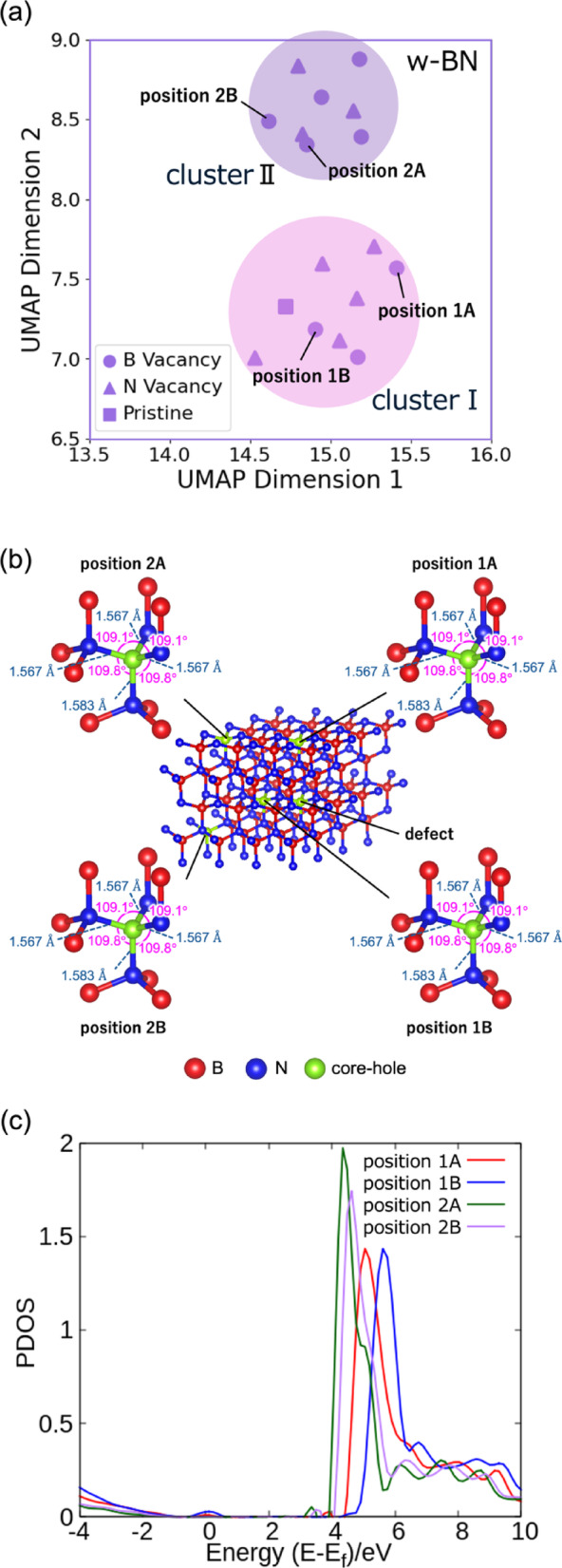
Atom projected total Density of States (DOS) for atoms in w-BN clusters I and II. (**a**) An enlarged view of the w-BN region in UMAP space, where the data segregate into two clusters (designated Cluster I and Cluster II) due to factors other than defect type. (**b**) Supercell structure showing the core-hole introduction positions (1A, 1B, 2A, and 2B) used in the calculations. The term “position” refers to the atomic positions where the core hole was introduced during the computation of XAS spectra and DOS. The bond distances and bond angles between the core hole and adjacent nitrogen atoms remained identical across all core-hole positions. (**c**) DOS of w-BN (VB) calculated at positions 1A, 1B, 2A, and 2B. XAS spectra computed with core holes at positions 1A and 1B belongs to Cluster I, whereas those at positions 2A and 2B were classified into Cluster II.

In the case of w-BN, the UMAP embedding separated the data into two distinct clusters (I and II) (Fig. 6a). Both clusters contained a mixture of V_B_ and V_N_ spectra, suggesting that the defect types were not distinctly segregated. However, inspection of the original crystallographic data revealed that both clusters shared identical local structural parameters: the B–N bond length along the c-axis is 1.583 Å, all other B–N bond lengths are 1.567 Å, the N–B–N bond angle along the c-axis is 109.8°, and the remaining N–B–N bond angles are 109.1° in both clusters (Fig. 6b). This indicates that the local structures in the vicinity of the defects are essentially identical, implying that the clustering arises from factors beyond mere geometric differences.

To further elucidate this discrepancy, we focused on the atom-projected total Density of States (DOS) of the core-hole atom to compare clusters I and II in detail. Figure 6c

presents the PDOS for the V_B_ configurations in the two clusters. The DOS was computed for the excited states of atoms including the core hole. Spectra obtained by introducing the core hole at positions 1A and 1B were assigned to cluster I, whereas those from positions 2A and 2B fell into cluster II. A comparison of the atom-projected DOS from these four positions revealed that the intensity of antibonding states is lower in cluster I than in cluster II. This trend was consistently observed even in the DOS calculated for atoms other than those at positions 1A–2B, suggesting that UMAP detect even the slightest differences in the electronic state. The observed variations in DOS intensity are likely attributable to subtle electronic effects, such as charge redistribution, rather than differences in bonding strength.

Furthermore, a comparison of the Fermi levels between the two clusters revealed that all atoms in cluster I exhibit lower Fermi levels than those in cluster II. This finding demonstrates that UMAP successfully clusters key physical quantities derived from the XAS spectra. Collectively, these results indicate that UMAP can clearly resolve even minor differences in the electronic states of BN. In essence, UMAP not only clusters the XAS spectra according to crystal structure and defect type but also discriminates based on subtle differences in charge transfer, as reflected in the DOS. These observations underscore the utility of UMAP for analyzing of complex spectroscopic data, suggesting that it can be a powerful tool for elucidating both structural and electronic nuances in advanced materials.

**Fig. 7:**
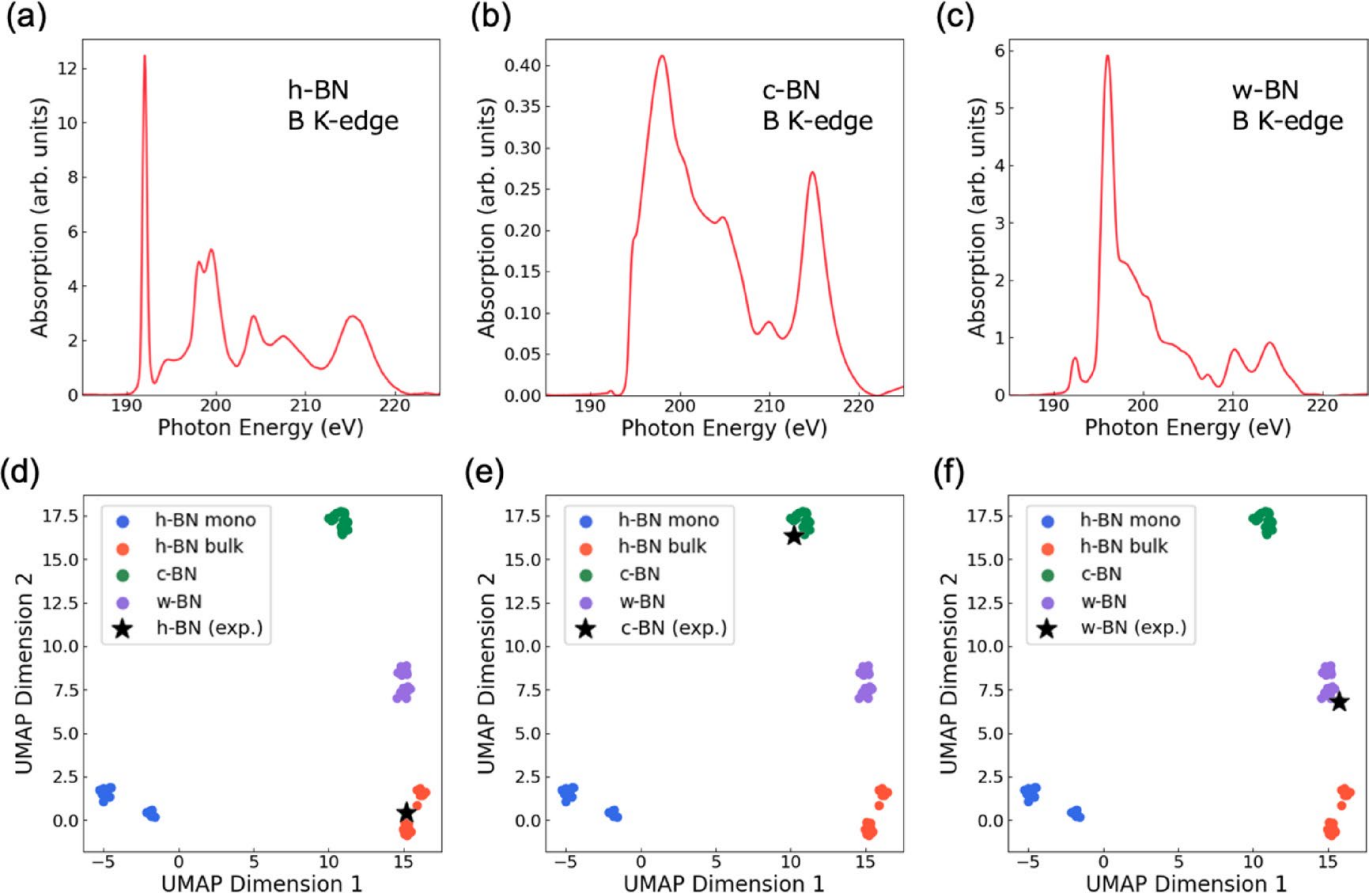
UMAP projection of experimental K-edge XAS spectra for three BN phases (h-BN, c-BN, and w-BN) onto the UMAP space derived from computed spectra. (**a**–**c**) Background corrected and resolution-aligned experimental spectra. (**d**–**f**) UMAP projections showing close alignment between experimental spectra and their respective theoretical clusters. The observed correspondence demonstrates that essential spectral features are preserved despite experimental noise and resolution differences, highlighting the potential of this integrated framework for autonomous, data-driven material identification.

To further validate the applicability of our framework to experimental spectra, we incorporated experimental XAS data for three BN phases: h-BN, c-BN, and w-BN (Fig. 7a–c). After applying step-function background correction and aligning the energy resolution (0.1507 eV), these spectra were projected into the UMAP space derived from the computed dataset (Fig. 7d–f).

For details of background correction, please refer to Section S4 in the Supplementary Information.

In all cases, the experimental spectra aligned closely with their respective theoretical clusters in the low-dimensional UMAP space, indicating that the essential spectral features were accurately captured despite differences in resolution and noise. This strongly supports the utility of our approach in identifying materials across different phases using real experimental data. It also suggests that UMAP effectively captures the essential spectral features while robustly handling noise and minor variations introduced by experimental conditions.

These findings imply that UMAP has strong potential as a tool for experimental material identification, enabling the autonomous recognition of materials based on a computed spectral database. As an applied experimental approach, operando XAS measurements during catalytic reactions especially those producing large-scale datasets represent a promising direction. This method can be extended to the automatic analysis of catalytic reaction mechanisms and the identification of active sites, offering a powerful tool for data-driven exploration in heterogeneous catalysis.

However, distinguishing specific defect types remains challenging. Future work will focus on expanding the spectral database and refining data preprocessing techniques to enhance the robustness of this approach as an automated method for analyzing the properties of unknown samples. Overall, our framework provides a powerful bridge between experimental and theoretical investigations, potentially accelerating innovations in functional material design. With further validation and refinement, its implementation at beamlines and expansion to a broader range of elements could lead a versatile platform for automated spectral analysis.

## Methods

### Materials and computational methods

The crystal structures of h-BN (mp-629015), c-BN (mp-1639), and w-BN (mp-2653) were sourced from the Materials Project^50^ and fully optimized using the non-relativistic electron exchange and correlation functional, Perdew-Burke-Ernzerhof (PBE)^51^. The h-BN monolayer structure was constructed by removing one atomic layer from the bulk structure. For the bulk h-BN lattice, the Brillouin zone integration was performed with a Γ-centered k-point mesh of 6 × 6 × 1, as described in Methfessel-Paxton smearing^52^ with a width σ of 0.05 eV was employed, which is suitable for semiconducting systems. The k-point meshes were adjusted for the h-BN monolayer, c-BN, and w-BN to 10 × 10 × 1, 6 × 6 × 6, and 6 × 6 × 4, respectively. A plane-wave energy cutoff of 500 eV was set, and convergence criteria for total energy and force per ion were set to approximately 10⁻⁸ eV and 0.005 eV/Å, respectively. To avoid intermolecular interactions between adjacent layers, a vacuum of 15 Å was applied for both h-BN structures. For detailed structural parameters, please refer to Fig. S7 and Fig. S8 in the supplementary section.

The supercell core-hole (SCH) method implemented in VASP was used to calculate the XAS for all the crystal systems investigated. For this purpose, a 4 × 4 × 2 supercell was utilized for the h-BN bulk, and a 6 × 6 × 1 supercell for the h-BN mono, a 3 × 3 × 2 supercell for the c-BN, a 4 × 4 × 2 supercell for the w-BN. For the PBE-level XAS calculations, a dense k-point mesh of 6 × 6 × 1 was used for the h-BN bulk, while the meshes were adjusted to 10 × 10 × 1 for h-BN mono, 6 × 6 × 6 for c-BN, and 6 × 6 × 4 for w-BN, respectively, along with 2000 empty bands, was used to ensure accurate results. The usefulness of the XAS approach in generating accurate energy features for atomic edges of supercell systems has been discussed elsewhere^53^. The Projector Augmented Wave (PAW) method^54^ with standard potentials was utilized for all atoms in calculations. The effect of spin-orbit coupling is not considered since the chemical systems investigated do not comprise heavy atoms. All calculations were performed using the Vienna Ab initio Simulation Package (VASP)^55,56^.

For each of the four supercell structures, two types of point defects were introduced: boron vacancies (V_B_) and nitrogen vacancies (V_N_), both modeled in their non-passivated states. In the case of the pristine structures, a single core hole was introduced, whereas for the defect structures, eight distinct core hole positions, relative to the location of the defects, were evaluated. Consequently, for each of the h-BN bulk/monolayer, c-BN, and w-BN structures, a total of 17 XAS spectra were obtained: one for the pristine structure, eight for the boron vacancy defect, and eight for the nitrogen vacancy defect. This resulted in a total of 68 XAS spectra, with each spectrum consisting of 266 data points, creating a high-dimensional dataset suitable for further analysis.

### Machine learning methods

To visualize the intrinsic data structure within the high-dimensional XAS spectral data and to highlight nontrivial differences in crystal systems and atomic defects, we applied dimensionality reduction techniques based on manifold learning. This approach rests on the hypothesis that high-dimensional data lie on a low-dimensional, nonlinear manifold, allowing the essential structure of the data to be preserved during dimensionality reduction. Embedding the data onto such a manifold facilitates visualization and subsequent analysis by maintaining the interrelationships among data points.

In our study, each XAS spectrum consisted of 266 data points, and a total of 68 spectra were analyzed, resulting in an extremely high-dimensional dataset. Dimensionality reduction was applied to project these data into a two-dimensional space. To achieve this, we employed both linear and nonlinear techniques. Principal Component Analysis (PCA) was used as the representative linear method, while Multi-Dimensional Scaling (MDS), t-distributed Stochastic Neighbor Embedding (t-SNE), and Uniform Manifold Approximation and Projection (UMAP) were utilized as nonlinear methods. Although previous studies have explored dimensionality reduction approaches that combine XAS with X-ray fluorescence (XRF) data^57^, our focus is solely on the XAS spectra, in anticipation of future experimental applications.

The implementations and settings for each method are detailed as follows. PCA, MDS, and t-SNE were implemented using the Python scikit-learn library, whereas UMAP was implemented via the umap-learn library. In the case of MDS, cosine similarity was adopted as the distance metric. For t-SNE, default hyperparameters were used except for setting the perplexity to 30, the learning rate to 200, and the number of iterations (n_iter) to 1000; Euclidean distance was employed as the metric. For UMAP, the principal hyperparameters were set to n_neighbors = 15 and min_dist = 0.1, with Euclidean distance also serving as the metric. All other parameters were retained at their default settings.

By applying these dimensionality reduction techniques, we embedded the high-dimensional XAS spectral data into a two-dimensional space and compared the performance of each method. This comparative analysis enabled us to identify the technique that most effectively visualizes nontrivial variations in crystal systems and atomic defects derived solely from the XAS spectra.

## Conclusion

In this study, we generated X-ray absorption spectra (XAS) from first-principles calculations for representative boron nitride (BN) structures including h-BN, c-BN, and w-BN as well as their analogues with point defects. By applying the unsupervised machine learning technique Uniform Manifold Approximation and Projection (UMAP), we successfully classified the complex spectral data according to crystal structure, defect type, and structural dimensionality. Notably, our analysis enabled the automated resolution of slight charge transfer differences from the XAS spectra, revealing nontrivial variations in electronic states, an approach not previously applied to light elemental periodic systems like BN.

UMAP demonstrated enhanced effectiveness over conventional dimensionality reduction methods such as Principal Component Analysis (PCA) and Multidimensional Scaling (MDS), particularly in handling nonlinear and high‐dimensional spectral data. Furthermore, by applying the computationally derived model to experimental h-BN XAS spectra, we validate its effectiveness in accurate structural identification.

In the future, this approach holds promise for extension to other material systems and the analysis of less characterized samples, paving the way for a versatile, automated tool for materials characterization. The influence of data point variations and distance metrics on dimensionality reduction outcomes remains uncertain, suggesting a potential direction for future investigation. This work underscores the power of integrating computational and experimental XAS data analysis, contributing to innovative materials design and advanced property evaluation.

## Supplementary Information

Below is the link to the electronic supplementary material.

Supplementary Material 1

## Data Availability

The primary data supporting the findings of this study are available within the article and its supplementary information. Additional data can be obtained from the corresponding authors upon reasonable request.

## Code availability

The codes used in this study are available from the corresponding authors upon reasonable request.
