# Supplementary material for: Automated elucidation of crystal and electronic structures in boron nitride from X-ray absorption spectra using uniform manifold approximation and projection
Source: Sci Rep. 2025 Nov 10;15:37736. doi: 10.1038/s41598-025-18580-z (PMC12603293; doi:10.1038/s41598-025-18580-z)
Supplement: Supplementary file 1 — Supplementary Material 1 [file 41598_2025_18580_MOESM1_ESM.docx]

**Automated Elucidation of Crystal and Electronic Structures in Boron Nitride from X-ray Absorption Spectra Using Uniform Manifold Approximation and Projection**

Reika Hasegawa^1^, Arpita Varadwaj^1*^, Alexandre Lira Foggiatto^1^, Masahito Niibe^2^, Takahiro Yamazaki^1^, Masafumi Horio^2^, Yasunobu Ando^3^, Takahiro Kondo^4^, Iwao Matsuda^2^, Masato Kotsugi^1*^

^1^Department of Material Science and Technology, Tokyo University of Science, 6-3-1, Niijuku, Katsushika, Tokyo, 125-8585, Japan

^2^Institute for Solid State Physics, The University of Tokyo, Kashiwanoha 5-1-5, Kashiwa, Chiba, 277-8581 Japan

^3^Institute of integrated research, Institute of Science Tokyo, Yokohama, Kanagawa, 226-8501, Japan

^4^ Institute of Pure and Applied Sciences, University of Tsukuba, Tsukuba, Ibaraki 305-8573, Japan

*Corresponding author’s e-mail: avaradwaj@rs.tus.ac.jp

**Electronic Supplementary Information (ESI)**

**S1. Eigenvectors of PCA**

　 The eigenvectors of the first and second principal components of the 68 XAS spectra obtained by SCH calculation and dimension-reduced by PCA are shown in Fig. S1, indicating that the energy bands of the σ coupling play a major role in both the first and second principal components of PCA. On the other hand, the energy region of the π-coupling peak has almost no effect on the principal components. This is consistent with the result that c-BN and w-BN, which are mainly characterized by the σ* peak, can be clustered, but h-BN bulk and monolayer, which are also characterized by the π* peak, cannot be clustered. This indicates that PCA fails to capture the π bonding features and only captures some of the features in the complex spectrum of BNs.


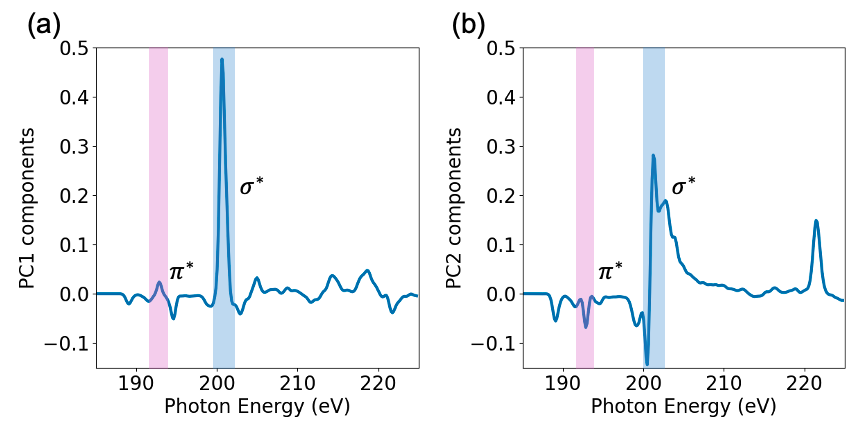
 Fig. S1. Eigenvectors of PC1 and PC2. (a) and (b) are the eigenvectors of PC1 and PC2, respectively. Both PC1 and PC2 have a large contribution from the σ* peak around 200 eV, while both have little contribution from the π* peak band around 190 eV. This means that PCA is losing information contained in the π* peak.

**S2. Differences in defect-adjacent XAS spectral shape**

　 The results of XAS spectra calculated by SCH calculations with core-holes in h-BN bulk, h-BN monolayer, c-BN, and w-BN supercells are shown. The XAS spectra calculated with core-holes in the atoms adjacent to the defects, indicated by the dotted lines, show larger changes in peak intensity, peak shift, and overall spectral shape than the other spectra. This indicates that atomic defects have a significant effect on the electronic structure.

t-SNE failed to achieve desired classification of each crystal structure. As shown in Fig. S2, the misclassified XAS spectra is originated from the atoms adjacent to defects. This spectral shape significantly different compared to other spectra. Hence, t-SNE is unable to accurately classify such slight electronic structural difference.


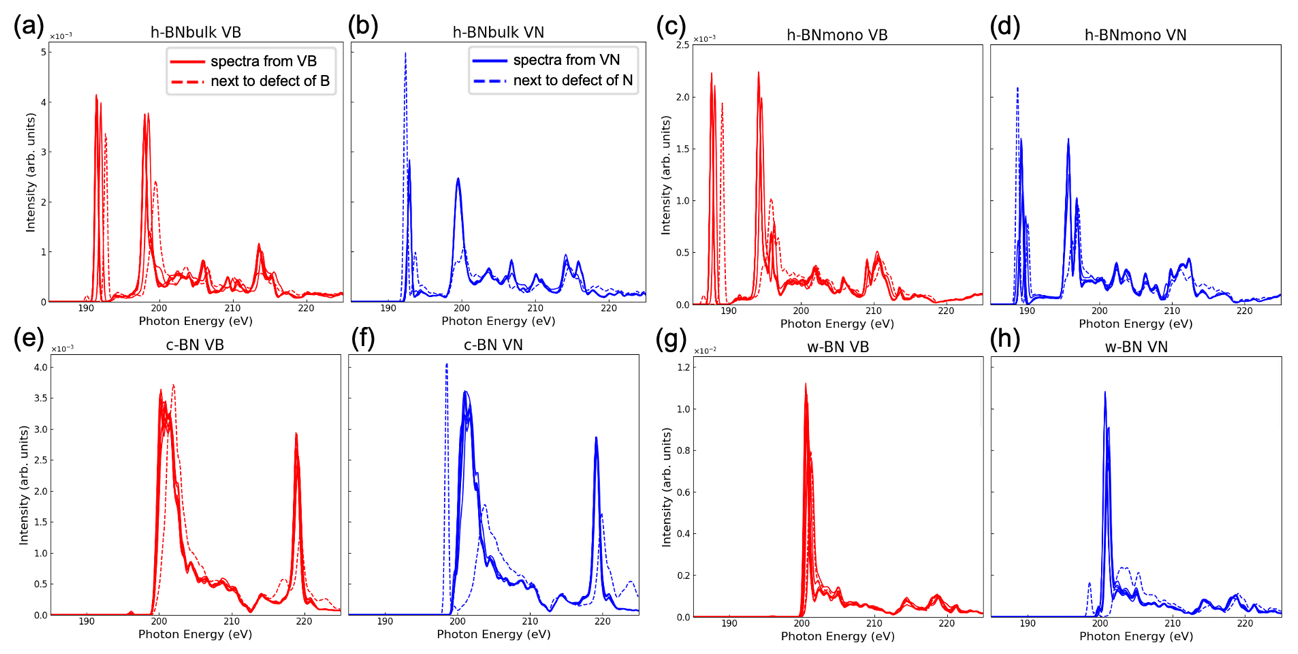


Fig. S2. XAS spectra calculated from each crystal system by SCH calculation.

XAS spectra of (a) h-BN bulk, (b) h-BN monolayer, (c) c-BN, and (d) w-BN. XAS spectra with core-hole introduced into atoms adjacent to defects are shown by dotted lines. For most of the structures, the dotted line spectra show a different behavior from the solid line spectra, and changes in peak intensity and peak shifts were observed.

**S3. Difference in spectra depending on the type of defect**

　 Finally, the spectra of V_B_, V_N_, and pristine were compared (Fig. S3). h-BN showed a shift of V_N_ toward higher energy than V_B_. The spectra of c-BN also showed a similar trend, also slightly, compared to h-BN. w-BN showed a very small difference in spectra depending on the type of defects compared to the other structures. These are consistent with the clear clustering of h-BN by defect in UMAP, the relative lack of clarity of the boundary between V_B_ and V_N_ in c-BN, and the lack of clustering by defect type in w-BN. In other words, it is shown that UMAP can capture even slight shifts in spectral shape and changes in peaks.


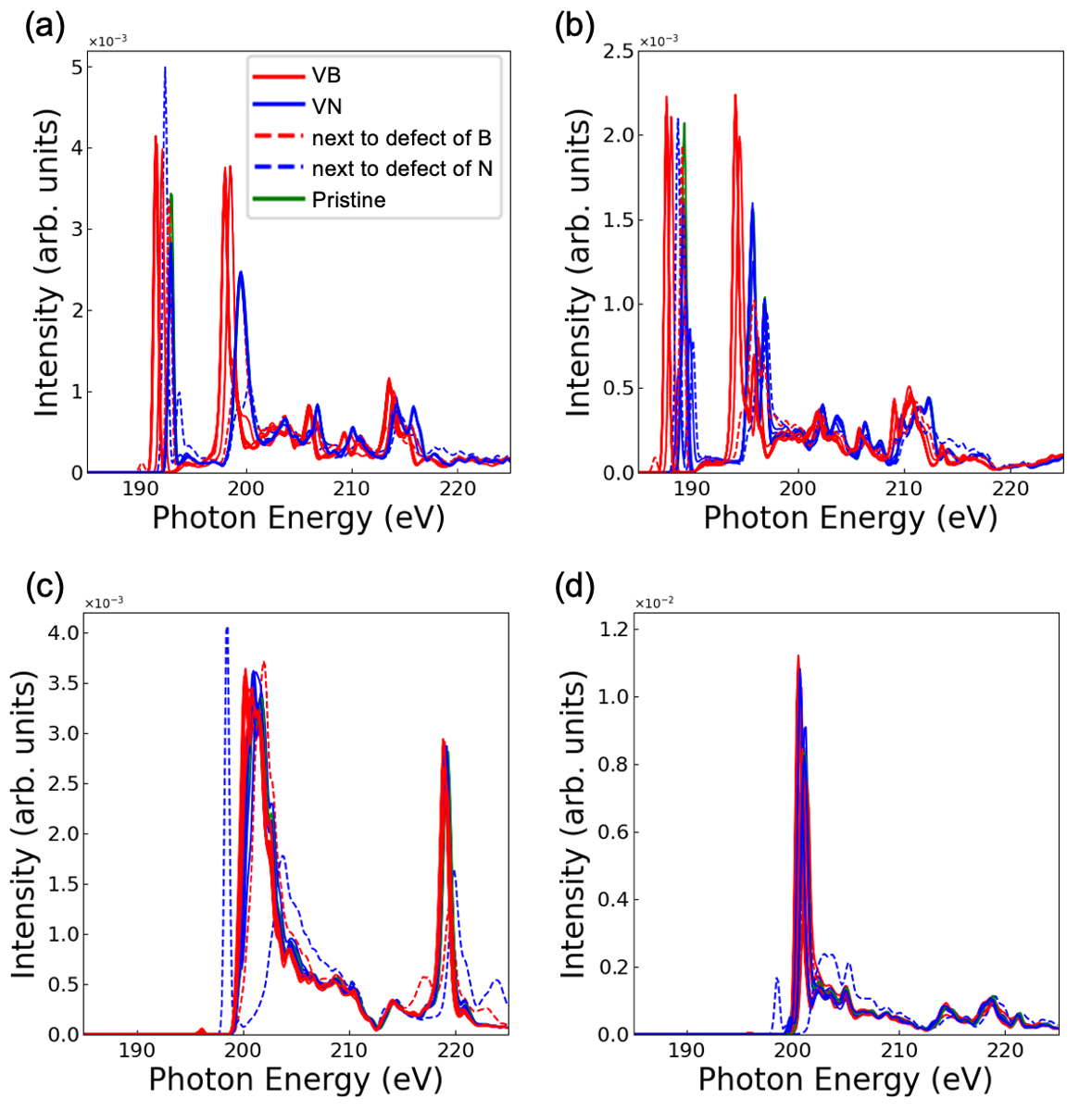


Fig. S3. Comparison of V_B_, V_N_ and pristine spectra. (a) is h-BN bulk, (b) is h-BN mono, (c) is c-BN, and (d) is w-BN; V_B_ spectra are shown in red, VN spectra in blue, and pristine spectra in green. h-BN clearly shows the difference between V_B_ and V_N_, while for w-BN, no V_B_ and V_N_ based spectra were not observed for w-BN. A slight peak shift between V_B_ and V_N_ was observed for c-BN.

**S4. Preprocessing of experimental spectrum**

Prior to applying UMAP to the experimental spectrum, preprocessing steps including smoothing and background correction were performed. In this process, a simple moving average (SMA) method was initially used to smooth the spectrum. For each data point, the average of five adjacent points (two before, one at, and two after the point) was calculated and used to replace the original value. The original data for the first and last two points were retained without modification.

Subsequently, background correction was applied using a step function. In case of h-BN the maximum slope of the spectrum in the vicinity of the σ* peak occurred at 197.6 eV. Therefore, the spectrum was divided into two regions, one before and one after this value. The region below 197.6 eV was fitted using the first step of the step function, while the region above 197.6 eV was fitted with the second step. For the first step, a horizontal line was defined such that the minimum absorption intensity below 197.6 eV was set to 0. Similarly, for the second step, a horizontal line was defined so that the minimum absorption intensity above 197.6 eV was also 0. To ensure a smooth transition between the two functions, a smooth step function based on the Gaussian error function (erf function) was applied. The background correction was then achieved by subtracting the constructed step function from the experimental spectrum (Fig. S4(b)).

For the experimental spectrum of c-BN, an upward-sloping background was observed. Accordingly, the first segment of the step function was defined as a sloped line such that the intensity before the σ* peak became zero. The second segment was defined as a semi-infinite line starting from the inflection point of the σ* peak at 193.06 eV and passing through the post-edge inflection point at 221.73 eV. Background correction was then performed in the same manner as for h-BN.

For w-BN, a four-segment step function was used. The first segment was a horizontal line passing through the minimum in the pre-edge region, with intensity set to zero. The second segment was also horizontal and spanned from the inflection point of the σ* peak at 196.64 eV to the post-edge inflection point at 206.89 eV. This was followed by a third horizontal segment across the fine structure region from 206.89 eV to 217.80 eV. Background subtraction was performed using this piecewise function.

To test the background subtraction dependensy in UMAP embeddings, we applied alternative step functions to the h-BN spectrum: (a) a simple horizontal line, (b) one-segment step, (c) a two-segment steps, and (d) a slope function (Fig. S4(a-d)). The corresponding UMAP results (Fig. S4(e)) showed that while minor spectral shape changes occurred, the embeddings shows reasonable dispersion to identify different crystal structure (see Fig. S4(e) for h-BN). Influence of background subtraction could be negligible for this crystallographic classification. Identification of defect type and concentration on experimental sample are beyond the scope of this study and open for future work.


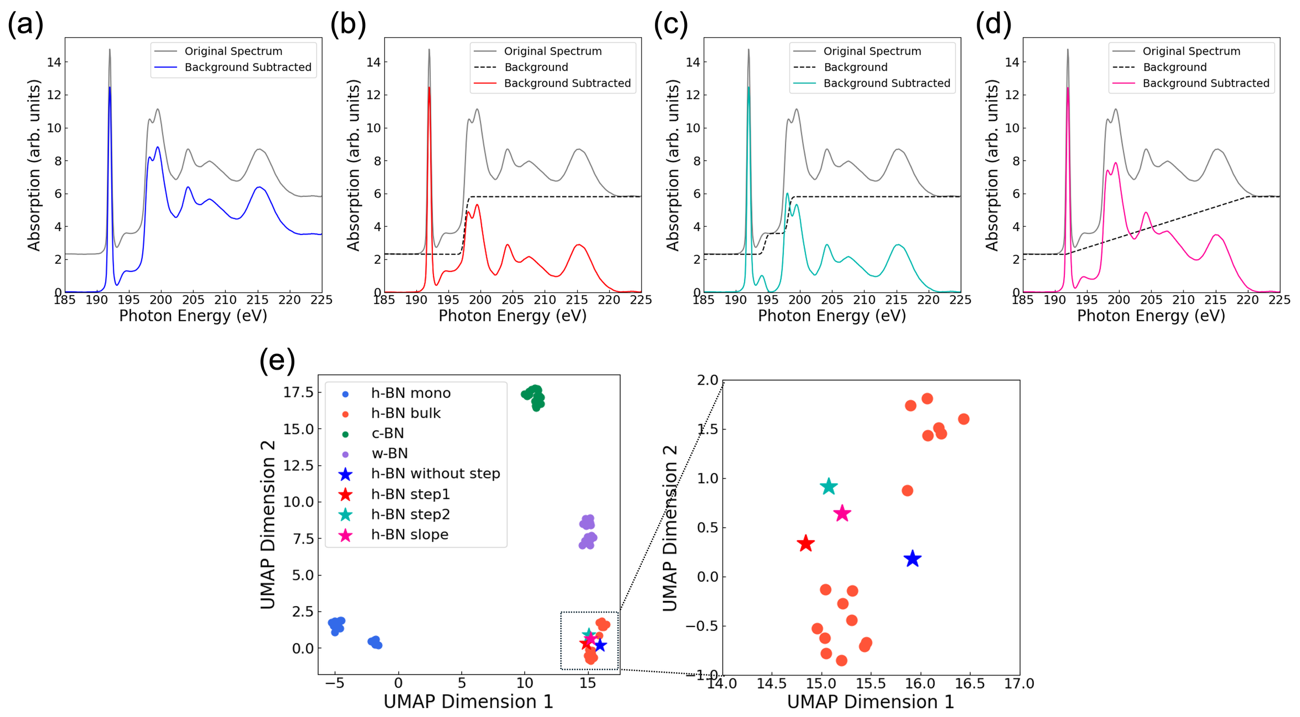


Fig. S4. Background correction methods applied to the h-BN experimental spectrum and corresponding UMAP embeddings. All spectra were smoothed using a simple moving average (SMA) prior to background subtraction. (a) Original spectra with horizontal background. (b) One-segment step background applied to the entire spectrum. (c) Two-segment step function applied to the entire spectrum. (d) Slope function applied to the entire spectrum. (e) UMAP embeddings of BN spectra including h-BN experimental data and right side is enlarged map of h-BN with different background dependencies.

**S5. PCA contribution ratio & PC3**

The contribution ratios and cumulative contribution ratios up to the sixth principal component were evaluated (Fig. S5). The cumulative contribution of PC1 through PC3 reached approximately 0.795. Based on this, a three-dimensional PCA plot was constructed using these first three components. However, no distinct clustering corresponding to different crystal structures was observed.

While a cumulative contribution of around 80% is typically considered sufficient for effective data representation in PCA-based analyses, the lack of clear structural separation even with PC3 indicates that PCA fails to capture the spectral variations essential for differentiating BN structures. This outcome suggests that PCA, being a linear dimensionality reduction method, may be inherently limited in resolving the complex, nonlinear relationships present in XAS spectra.


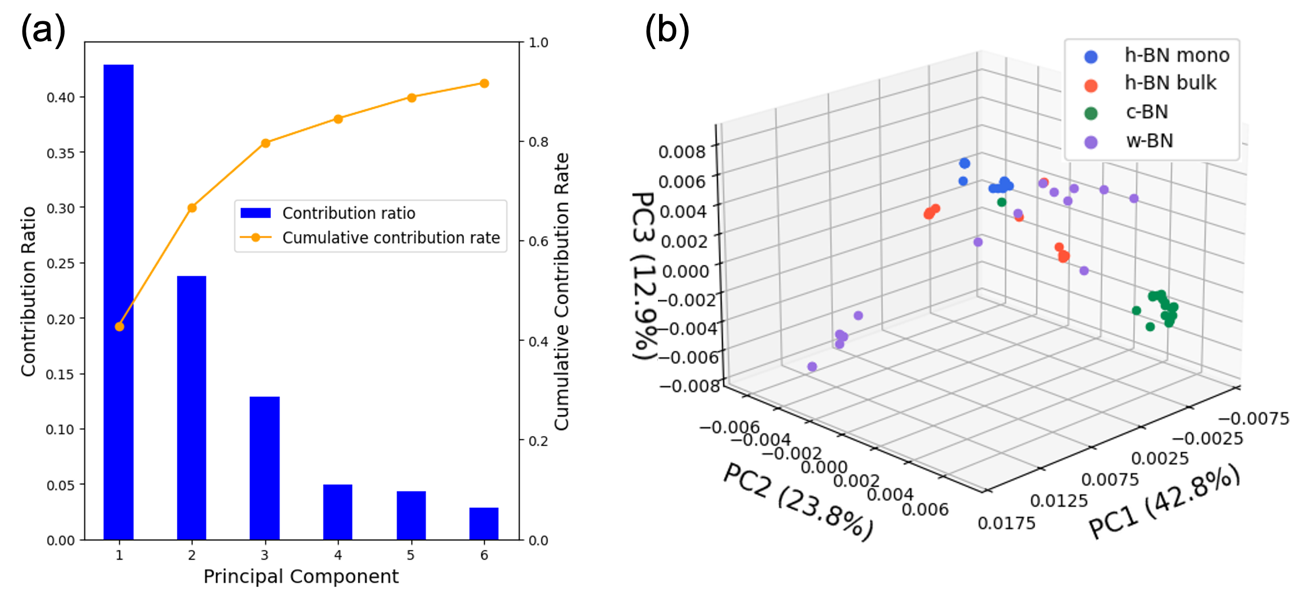


Fig. S5. (a) Contribution ratios and cumulative contribution ratios of each principal component obtained from PCA. The cumulative contribution of PC1 to PC3 reaches 0.795. (b) A 3D PCA plot constructed using the first three principal components. Despite capturing the majority of the variance, no clear clustering based on crystal structure is observed, suggesting that PCA may be limited in its ability to distinguish structural differences in XAS spectra.

**S6. Hyper parameter of UMAP & t-SNE**

Hyperparameter optimization was conducted for both t-SNE and UMAP. For t-SNE, the *perplexity* parameter, which relates to the effective number of nearest neighbors, must be carefully tuned according to dataset size, while the *learning rate* influences convergence and can lead to unstable embeddings if set too high. Considering these factors, we selected *perplexity* and *learning rate* values that provided the clearest inter-cluster separations, but even then, t-SNE’s reproducibility remained lower than that of UMAP.

For UMAP, the key hyperparameters are *n_neighbors* and *min_dist*. The *n_neighbors* parameter controls the balance between local and global structure preservation; smaller values emphasize local neighborhoods, while larger values capture broader, global relationships. The *min_dist* parameter determines how tightly data points are clustered in the low-dimensional space; smaller values result in more compact clusters, while larger values spread points further apart.

As shown in Fig. S6, tuning these parameters did not significantly alter the overall clustering topology for UMAP, indicating that UMAP provides stable embeddings across a wide hyperparameter range. Given the objective of extending this approach to automated analysis of experimental data, we chose hyperparameters that emphasize local structures, which enhances sensitivity to subtle variations such as atomic defects and facilitates reliable automatic clustering.


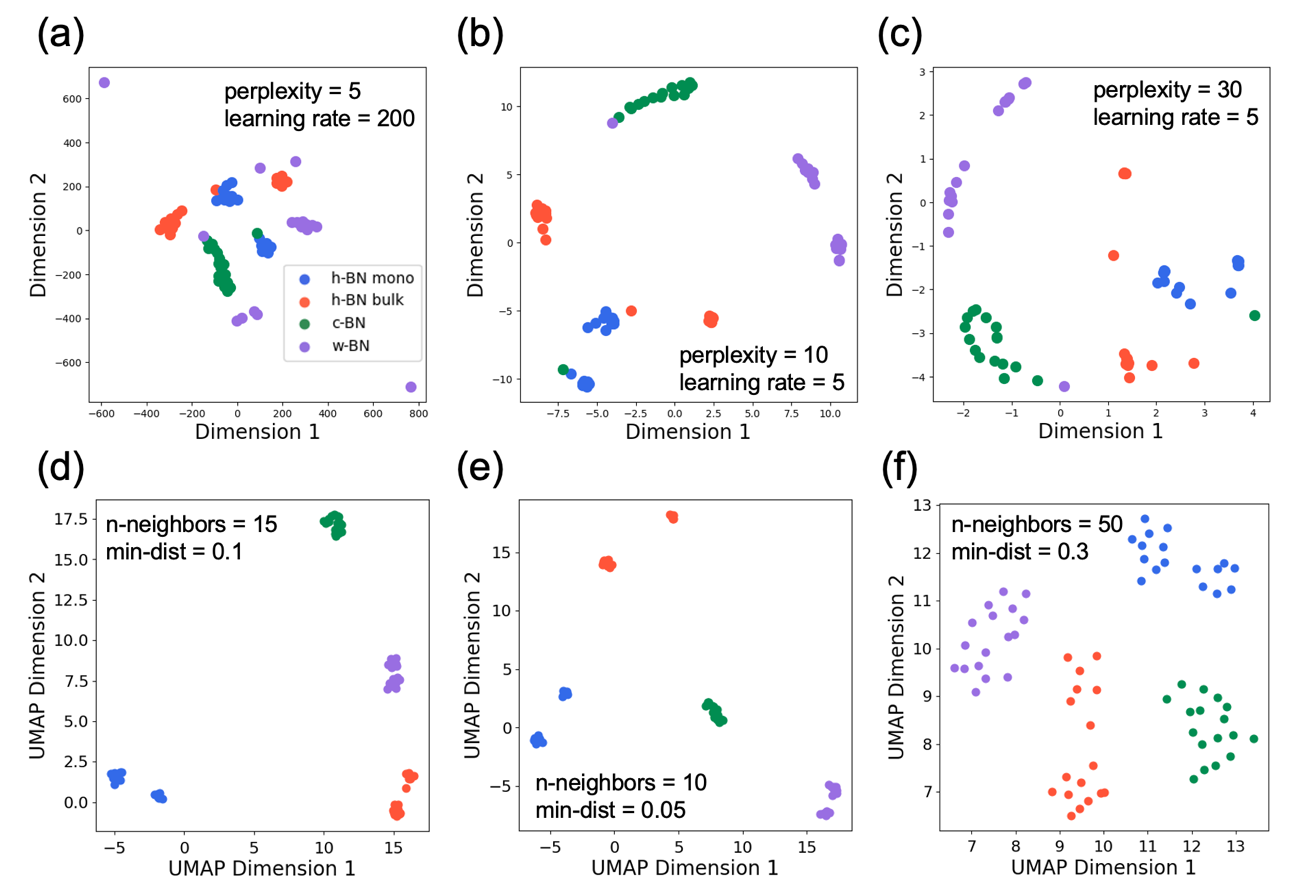


Fig. S6. Comparison of t-SNE and UMAP embeddings under different hyperparameter settings. The upper panel ((a)–(c)) shows t-SNE plots where *perplexity* and *learning rate* were varied from their default values. The lower panel ((d)–(f)) presents UMAP plots with varying hyperparameters: (d) default settings, (e) parameters emphasizing local structure (smaller *n_neighbors*), and (f) parameters prioritizing global structure (larger *n_neighbors*). In all cases, t-SNE did not produce distinct clusters comparable to UMAP. The specific hyperparameter values for each plot are indicated in the insets.


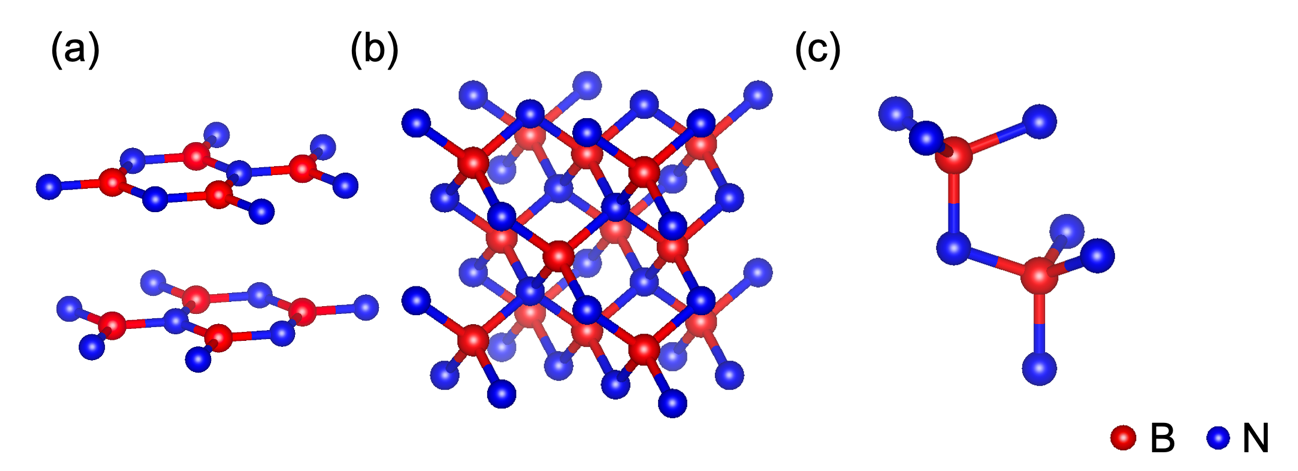


Fig. S7. PBE optimized polymorphs boron nitride (a) unit cell of bulk h-BN, (b) unit cell of c-BN，(c) unit cell of w-BN.

Table S1: PBE optimized lattice constants (a, b, c, a, b, and g), and energy/atom for different polymorphs of boron nitride. Values are given in angstrom and degrees.

| Structure | Material Project ID | Space group | a (Å) | b (Å) | c (Å) | α (°) | β (°) | γ (°) | Energy/ Atom (eV) |
| --- | --- | --- | --- | --- | --- | --- | --- | --- | --- |
| h-BN | mp-629015 | P6_3/mmc | 2.502 | 2.502 | -- | 90 | 90 | 120 | -8.792 |
| c-BN | mp-1639 | F-43m | 3.626 | 3.626 | 3.626 | 90 | 90 | 90 | -8.726 |
| w-BN | mp-2653 | P6_3mc | 2.553 | 2.553 | 4.227 | 90 | 90 | 120 | -8.708 |


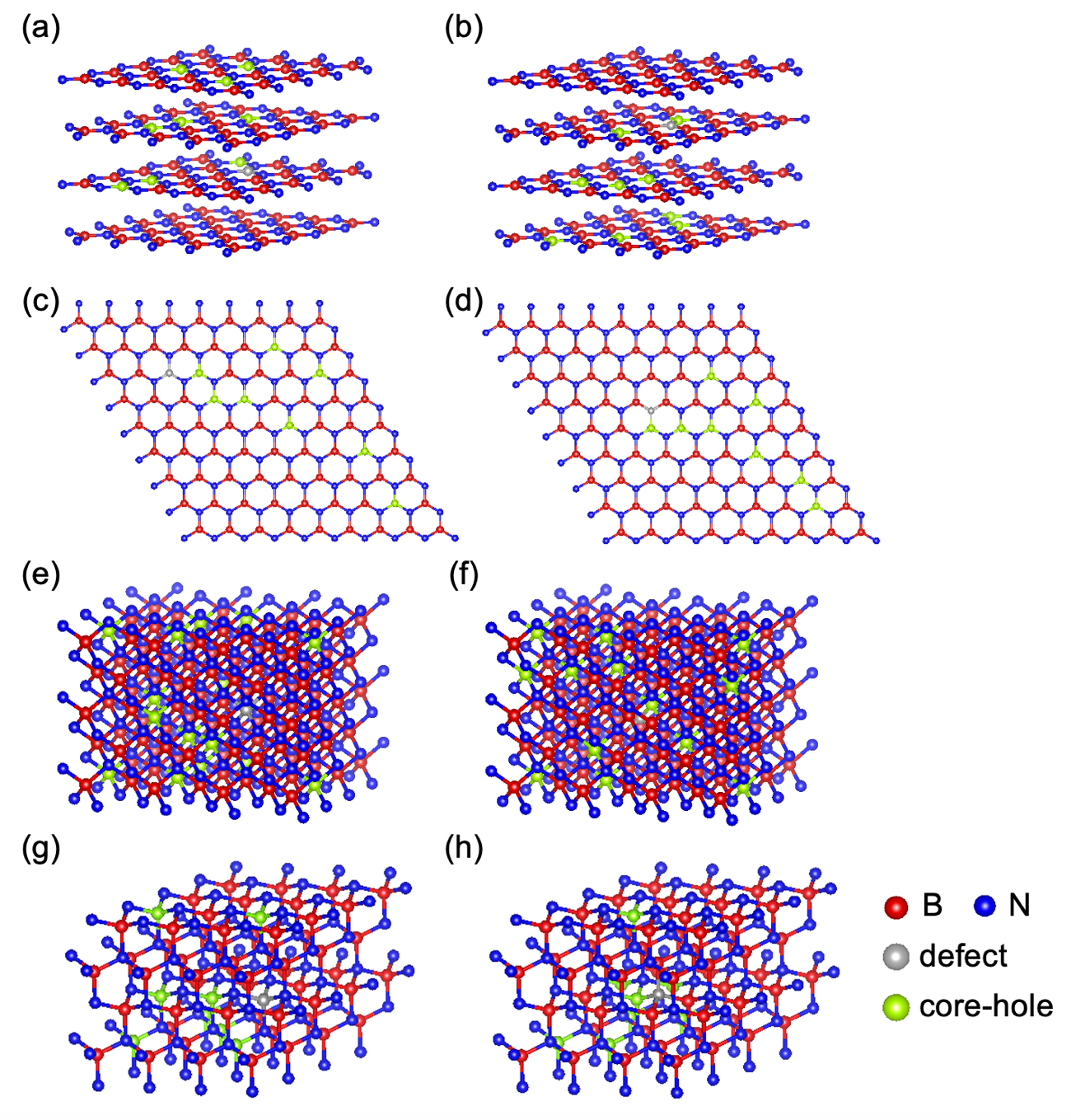


Fig. S8. Positions of point defects added to the supercell for each structure along with the core-hole position introduced during the SCH calculation. (a) h-BN bulk (V_B_), (b) h-BN bulk (V_N_), (c) h-BN monolayer (V_B_), (d) h-BN monolayer (V_N_), (e) c-BN (V_B_), (f) c-BN (V_N)_, (g) w-BN (V_B_), (h) w-BN (V_N_). The positional relationship between the core hole and defects varies for each structure
